# Does cattle and sheep grazing under best management significantly elevate sediment losses? Evidence from the North Wyke Farm Platform, UK

**DOI:** 10.1007/s11368-021-02909-y

**Published:** 2021-03-13

**Authors:** S. Pulley, L. M. Cardenas, P. Grau, S. Mullan, M. J. Rivero, A. L. Collins

**Affiliations:** 1 Sustainable Agriculture Sciences, Rothamsted Research, North Wyke, Okehampton, Devon EX20 2SB UK; 2 Bristol Veterinary School, University of Bristol, Langford, Somerset BS40 5DU UK

**Keywords:** Sediment yield, Grazing livestock, Soil damage, Livestock management, Stocking rate

## Abstract

**Purpose:**

Intensive livestock grazing has been associated with an increased risk of soil erosion and concomitant negative impacts on the ecological status of watercourses. Whilst various mitigation options are promoted for reducing livestock impacts, there is a paucity of data on the relationship between stocking rates and quantified sediment losses. This evidence gap means there is uncertainty regarding the cost–benefit of policy preferred best management.

**Methods:**

Sediment yields from 15 hydrologically isolated field scale catchments on a heavily instrumented ruminant livestock farm in the south west UK were investigated over ~ 26 months spread across 6 years. Sediment yields were compared to cattle and sheep stocking rates on long-term, winter (November–April), and monthly timescales. The impacts of livestock on soil vegetation cover and bulk density were also examined. Cattle were tracked using GPS collars to determine how grazing related to soil damage.

**Results:**

No observable impact of livestock stocking rates of 0.15–1.00 UK livestock units (LU) ha^−1^ for sheep, and 0–0.77 LU ha^−1^ for cattle on sediment yields was observed at any of the three timescales. Cattle preferentially spent time close to specific fences where soils were visually damaged. However, there was no indication that livestock have a significant effect on soil bulk density on a field scale. Livestock were housed indoors during winters when most rainfall occurs, and best management practices were used which when combined with low erodibility clayey soils likely limited sediment losses.

**Conclusion:**

A combination of clayey soils and soil trampling in only a small proportion of the field areas lead to little impact from grazing livestock. Within similar landscapes with best practice livestock grazing management, additional targeted measures to reduce erosion are unlikely to yield a significant cost-benefit.

**Supplementary Information:**

The online version contains supplementary material available at 10.1007/s11368-021-02909-y.

## Introduction

An increase in soil erosion rates due to modern intensive agriculture has been identified as a major cause of the degraded ecological status of freshwaters (Novotny 1999; Foley et al. 2005; Kemp et al. 2011). Whilst recently cultivated soils have been shown to be the most important sediment source in most temperate agricultural catchments (Walling and Collins 2005; Walling et al. 2008), grasslands are the dominant land use in many catchments and have also been shown to impact water quality negatively where intensive ruminant farming is undertaken (Heathwaite et al. 1990; Hooda et al. 2001; Harrod and Theurer 2002).

It has been recognised that intensively managed grasslands are associated with damage to soils and therefore an increased risk of soil erosion when compared to natural or ungrazed grasslands (Bilotta et al. 2007). However, little quantitative data exists on the links between livestock and quantified soil erosion (Bilotta et al. 2007). Direct damage can be caused to soils through the impact of animal hooves exerting a shear stress and dislodging a layer of soil which is then susceptible to erosion by rainsplash and runoff (Alexandrou and Earl 1997). In addition, soil compaction influenced by animal weight and the relative area of the hoof can degrade the soil structure (Silva et al. 2003). Compaction results in a decrease in the void spaces between soil peds and therefore also a decrease in its hydraulic conductivity, resulting in a greater proportion of rainfall generating overland flow (Taylor 1971; Redmon 2002). This increased flow has the potential to detach and transport sediment particles. The susceptibility of a soil to compaction is determined by its physical properties such as texture, biota, water regime, and chemistry (Horn et al. 1995). For example, silt loam soils are more susceptible to compaction than sandy, fine-textured, or clayey soils (Horn et al. 1995). Soil moisture content is also a key control, with wet soils being more susceptible to compaction than dry soils (Gysi et al. 1999), apart from when soils are fully saturated with no air filled void spaces (Smith et al. 1997).

In addition to physical effects on soil, grazing and trampling also cause a loss of sward cover, which can increase the area of a field where soils are exposed to raindrop impact (Busby and Gifford 1981) and therefore the risk of soil erosion. For example, Sanjari et al. (2009) identified that a minimum 70% surface cover by vegetation was required to efficiently protect soil from erosion in the south-east region of Queensland, Australia. The loss of sward cover can also lead to soil crusting, decreasing its hydraulic conductivity and consequently increasing runoff and soil erosion (Duley 1939; Mcintyre 1958; Li et al. 2001).

Good soil structure with high sward productivity and without excessive runoff generation is a function of the stability of soil aggregates (Amézketa 1999). As such, aggregate stability has been identified as a key indicator of soil health (Arshad and Coen 1992). Aggregate disintegration has been linked to multiple factors such as raindrop impact (Shainberg et al. 1992), pH (Keren et al. 1988), and electrolyte concentrations (Crescimanno et al. 1995). The trampling of soils by grazing animals has been linked to a decrease in aggregate stability in Alberta Canada, Texas USA, Western Australia, and British Columbia Canada (Johnston 1962; Warren et al. 1986; Proffitt et al. 1995; Broersma et al. 2000). However, Evans et al. (2012) found that moderate stocking rates (0.6 animal-unit months ha^−1^) over a 30-year period in a Canadian temperate grassland did not reduce the stability of soil aggregates suggesting that a causal link between livestock grazing and reduced aggregate stability is not present in all landscapes.

Rotational grazing systems were introduced in the 1960s with the aim of improving soil condition during scheduled periods when animals are excluded from fields (Holechek et al. 1999). It has been shown that periods of animal exclusion can reduce runoff and soil erosion when compared to continuous grazing (McGinty et al. 1979; Wood and Blackburn 1981; Warren et al. 1986; Sanjari et al. 2009). However, soil compaction has still been observed when rotational grazing has been used over extended time periods (Bryant et al. 1989; Dormaar et al. 1989). Other targeted management measures aimed at reducing soil erosion in grasslands include those aimed at improving general soil quality such as removing livestock from fields during very wet periods, leaving permanent or temporary buffer strips between grazing areas and watercourses, loosening compacted soils, reseeding unproductive grasslands, reducing stocking rates, and reducing the length of the grazing season (Newell Price et al. 2011). Within the UK, housing cattle indoors during winter months when soils are wet to avoid soil damage is considered good standard practice (DEFRA 2009). Certain mitigation measures can also be targeted based upon a visual assessment of soil condition such as frequently moving feeders or providing hard bases for water troughs when soils around them are visually heavily poached (Newell Price et al. 2011). At present, however, there is a paucity of field scale data on the changes in sediment yield which are associated with livestock grazing under best management. Instead, previous work has focussed on comparing best and worst case scenarios. As a result, the scope for delivering additional benefits by implementing policy preferred mitigation options such as periodically moving feeder rings, further reducing the length of the grazing day/length, further reductions in stocking rates and gateway re-siting (Newell Price et al. 2011), when best practice is already in place, is difficult to quantify. There remains a need to address this evidence gap, especially since visual inspections and audits of grazing livestock farms might result in unnecessary measures being recommended above and beyond the critical elements of best practice. Accordingly, this study compared sediment yields from 15 hydrologically isolated grassland fields on the North Wyke Farm Platform (NWFP) in south west England, to cattle and sheep stocking rates over ~ 26 months within a ~ 6-year (2013-2019) monitoring period in an attempt to quantify their effects on sediment losses within a best practice management regime. In so doing, the study also investigated the impacts of livestock on physical soil properties to provide supportive mechanistic understanding.

## Study area

The NWFP (50^∘^46^′^10^′′^N, -3^∘^54^′^05^′′^W) is in a lowland temperate landscape in the south west of the UK and is the most instrumented ruminant farm platform in the world (Orr et al. 2016). It experiences mean annual rainfall of 1053 mm. Topsoils include Hallsworth—a seasonally waterlogged clayey Dystric Gleysol, Halstow—a slowly permeable clayey Gleyic Cambisol and Denbigh a well-drained silty loam Brown Earth (Avery 1980). These overlay a poorly permeable stony clay subsoil which is heavily mottled. Topsoils have a clay content of approximately 36%, whilst subsoils have a corresponding content of approximately 60% (Harrod and Hogan 2008). These soils are representative of ~ 1843 km^2^ of temperate lowland ruminant grazing landscapes across England (Collins et al. 2021).

**Fig. 1:**
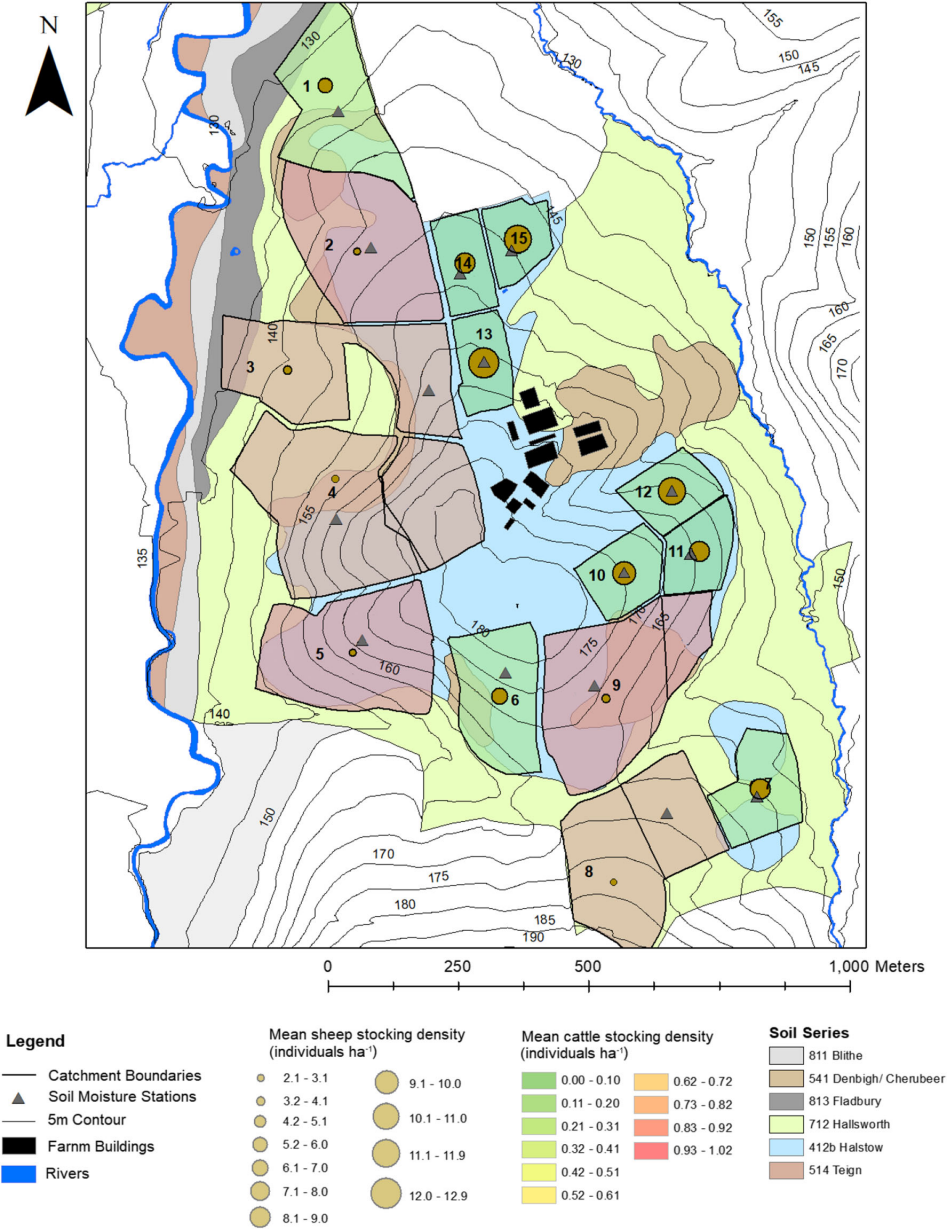
The NWFP with catchment numbers and livestock stocking rate, modified from Pulley and Collins (2019)

The NWFP consists of 15 hydrologically isolated field scale catchments which range in area from 1.54 to 7.75 ha (Fig. 1). The catchments have mean slopes of between 4.17 and 9.71 degrees at varying aspects around a central hilltop between the River Taw and its tributary Cocktree Stream. The NWFP operates experimentally as a commercial farm following best management practices. Its scientific purpose is to test the efficacy and sustainability of beef and sheep grazing systems (Orr et al. 2016; Takahashi et al. 2018). Accordingly, the 15 field scale catchments are divided into three farmlets which test sustainability trade-offs for each system. The three systems are: (1) business-as-usual long-term permanent pasture (BAU); (2) scheduled ploughing and reseeding for a high sugar grass monoculture (HSG); and (3) ploughing and reseeding for a HSG/clover mix (HSGC). Catchments under treatments 2 and 3 have been ploughed and re-seeded in four phases since the initiation of data collection on the NWFP in 2012. Prior to this ploughing, all the catchments were permanent pasture and had the same management and similar productivity (Orr et al. 2019). Thirty (mainly Charolais × Hereford-Friesian and Limousine × Hereford-Friesian, with gradual conversion to Stabiliser × Hereford-Friesian and Stabiliser breed from 2017 onwards) calves from an adjacent cow-calf enterprise are randomly assigned to each farmlet at the point of weaning in autumn at a mean wight of 418 kg. Cattle are normally housed from October to April to avoid structural degradation of seasonally waterlogged soils and then kept at pasture on their respective farmlet until reaching target weights of ~ 555 kg for heifers and ~ 620 kg for steers. Farmyard manure stored in middens during the winter housing period is used to fertilise the grazed pastures between silage cuts always going back to the same pasture that fed those animals. Suffolk × Mule ewes and their lambs sired by Charollais rams were assigned to each farmlet each spring (50 ewes in 2013 and 2014, and 75 ewes from 2016 onwards—until 2015 ewes were allocated randomly each spring, from 2016 onwards, ewes stayed in the same farmlet until culled when ewe lambs were added). With a lambing rate of 1.8–1.9, this results in a flock size of ~ 140–220 sheep across the entire farm platform until mid-autumn, when lambs reach a target weight of ~ 43.0 kg and are sold for slaughter. For the different fields on the NWFP, mean sheep stocking rates range from 0.15 to 1.00 UK grazing livestock units (LU) ha^−1^ (2.1–12.9 animals ha^-1^) and 0 to 0.77 LU ha^−1^ (0–1.02 animals ha^−1^) for cattle. Livestock units are defined as 0.75 for beef cattle, 0.11 for lowland ewes, and 0.04 for lambs under 1 year in age. Cattle were primarily present in the larger fields (catchments 2, 3, 4, 8, and 9) and sheep in the smaller fields (catchments 6, 7, 10, 11, 12, 13, 14, 15).

The grazing strategy at the NWFP is continuous (variable) stocking (grazing area is adjusted to maintain a target average sward surface height), with two silage cuts from selected fields (May and July each year). Grazing management on the NWFP is designed to follow best practice, wherein livestock are housed over the winter months when the soils are seasonally waterlogged and prone to excessive poaching and pugging; stocking rates are reduced during wet periods although to date this was only necessary from the 26 April to 12 May 2012 which was prior to the time period examined in this study, drinking troughs have hard bases (approximately 3 m × 1 m) to protect the immediately adjacent soils from trampling and exfoliation and ditches and streams are fenced off to prevent livestock access. A general overview of recommended good farming practice in the UK is provided by DEFRA (2009).

## Materials and methods

### Data collection

Water and sediment fluxes from the 15 field scale catchments were recorded at 15-min intervals between the 14/08/2013 and 14/01/2019. Each catchment is hydrologically isolated by a border of clean carbonate-free gravel filled French drains, which converge on a collection chamber where turbidity is recorded (YSI 6600 V2 multiparameter sonde; up to May–September 2016 and thereafter YSI EXO 2; Xylem Inc Rye Brook, NY, USA). The French drains consist of a perforated pipe positioned in a trench and surrounded by gravel. The purpose of the collection chambers was to ensure that the sondes did not dry out during periods of low rainfall since runoff at field scale is not continuous. The collection chamber then enters an open channel where discharge is measured using an OTT hydromet pressure transducer (OTT hydromet, Loveland, CO., USA) in an H-flume (TRACOM Inc., GA, USA) with the capacity for a 1 in 50-year runoff event.

Calibration of the multiparameter sondes for turbidity was conducted quarterly using solutions of 0 and 124 formazine nephelometric turbidity units (FNU). Turbidity was converted into suspended sediment concentration (SSC) using calibrations derived from the routine collection of water samples by automatic samplers (ISCO 3700, Teledyne ISCO). The retrieved 100-ml samples were filtered through 0.7-μm pore size glass fibre paper and oven dried at 105 °C for 60 min to quantify SSC. Measured turbidity and SSC were included in a linear regression to form the calibration shown in Eq. (1).

1 $$ SSC=1.1804\ast NTU+0.0472\ \left(\mathrm{r}2=0.75\right) $$

As turbidity during flows of less than 0.2 l s^−1^ was not measured routinely due to inadequate water depth, the intercept value of the SSC-turbidity relationships was used to infill these periods in the field discharge records (Pulley and Collins 2019). Rainfall was measured in the centre of each catchment at 15-min intervals using and an Adcon RG1 (Adcon, Austria) tipping bucket rain gauge with a 0.2 mm resolution. Soil moisture was also recorded at the same locations and interval at depths of 10, 20, and 30 cm using Adcon SM1 soil moisture stations.

The time series of livestock numbers and location were retrieved from the farm records stored in the Farm Platform Portal (https://nwfp.rothamsted.ac.uk/). Previously unpublished data generated on the NWFP as part of past research projects was used to gain an indication of the effects of livestock on the soils and their risk of erosion. Specifically, this included the extent to which cattle preferentially use different areas of each field and which proportion of the field soil was visibly damaged and bare of vegetation. The location of cattle in catchments 5 and 9 was recorded by attaching GPS tags (Bio-loggers constructed by Bangor University; Fehlmann and King 2016) based on the design of F2HKv2 tracking collars (Fehlmann et al. 2017). Monitoring in catchment 9 took place between the 15/05/2018 and 22/05/2018 and involved 24 cattle (out of 30 animals grazing in the field in that period). Monitoring in catchment 5 took place in the bottom half of the catchment between the 21/06/2018 and the 27/06/2018 for 18 cattle (out of 30 animals). The data collected during hours of darkness was not used in any analysis as the cattle were mostly stationary and lying down during these times. The percentage of soil area damaged by livestock and the total area of damaged soil (m^2^) were identified manually using NDVI calculated from a 5-cm resolution aerial photograph taken in mid-2016 in ARCGIS 10.5.

Soil bulk density was determined using a 10-cm deep and 6-cm diameter ring as part of the scheduled July 2016 spatial survey of the NWFP. Livestock management is not changed prior to the survey, and as such, stocking rates are high in some fields and low in others at the point of sampling (Supplementary Fig. 1). Sampling sites were positioned using a 25-m resolution grid which covered the whole farm platform. Bulk density was calculated by dividing the dry mass of soil by the core volume. The mass and volume of stones in each sample were subtracted prior to calculation. Stones were removed after drying the sample at 105 °C by disaggregating using a pestle and mortar and passing the samples through a 2-mm mesh. The mass of the > 2 mm fraction was recorded, and its volume was measured by placing it into a measuring cylinder and measuring the volume of water displaced. Additional details of the study site and sampling methods are provided in Orr et al. (2016) and Pulley and Collins (2019).

### Data analysis

The 15-min time series data was initially converted into total daily water flux, sediment flux, rainfall, and mean daily soil moisture content. The ~ 6-year daily time series produced were then plotted with the mean cattle and sheep stocking rates to observe any potential relationships between hydrology, the animal-to-land relationship, and sediment yield. Periods in which scheduled ploughing and reseeding took place and the subsequent autumn and winter months (until 31 March the next year) were not plotted since previous work (Pulley and Collins 2019) has already confirmed the substantial impact of these operations on sediment loss on the NWFP. Total sediment (excluding ploughed periods) yields (t ha^−1^ yr^−1^) were then calculated for the whole 6-year monitoring period and compared to hydrological factors such as total water flux and SSC in a Spearman correlation analysis to determine their primary controls.

Data was then excluded for any days where complete records were not available for all flumes. These periods comprised times when there were equipment failures (primarily for most of 2016) or during winters immediately after scheduled ploughing and reseeding, defined as up to 31 March in the following year. It was ensured that the datasets for every flume covered identical time periods which equated to 26.4 months of data. The re-calculated sediment yields were then compared to the mean number of individual sheep and cattle in each catchment and the mean stocking rate (LU ha^−1^) over the period to identify any impacts of livestock presence on long-term sediment yields.

The data was then analysed at a shorter monthly timescale. Monthly sediment yields were included in a Spearman rank correlation analysis with hydrological factors (water flux, mean SSC, rainfall, mean soil moisture) as well as sheep and cattle stocking rate. As soil damage caused by livestock during the summer and autumn grazing season may manifest as a higher sediment yield during the subsequent winter, a second analysis was conducted. Here, sediment yields were calculated between the 1 November and 31 of March for each year, to represent the periods when soil moisture is typically fully saturated and most erosion takes place. These yields were divided by water flux to account for inter-annual differences in rainfall. The mean stocking rates of sheep and cattle were calculated from the 1 April to the 31 of March for each flume and year, ending at the same date as the sediment yield calculation. The data covering the 2015–2016 winter was not used as equipment failure resulted in data for only the first half of this period being available, generating a disproportionately high calculated sediment yield as yield typically reduced over the course of the winter. Mean cattle stocking rate in October and November was then compared to the calculated water flux-normalised winter sediment yields to identify any impacts of leaving livestock out on saturated soils during autumn months. Sheep stocking rate during the October–March periods was also compared to the sediment yields as some animals were present in the fields (breeding ewes plus lambs not finished in summer-autumn) throughout the entire year, and there was therefore a potential risk of damage to soils during winter months. The October–November dates were selected based upon increasing soil wetness during this time and still some presence of cattle, although the best practice used on the FP meant that when soils became too wet cattle were bought indoors. The October–March date for sheep was selected as soils most often reached saturation in October and during March vegetation growth rates increased allowing for most of the previous year’s effects of livestock on sward cover to be reset. The latest date in which cattle were left within the fields was also included in this analysis as the presence of livestock when soils are wetter is likely to cause greater structural damage than when dry.

To determine the possible impacts of livestock on soil structural properties, the point density tool was used in ARC GIS 10.5 to calculate the percentage of time which the cattle spent in each cell of a grid of 10 × 10 m cells overlaid between the maximum and minimum *x* and *y* coordinates of each field. The areas of damaged soil lacking grass cover, as well as soil bulk density, were also mapped so that spatial patterns could be compared.

## Results

### Time series analysis

**Fig. 2:**
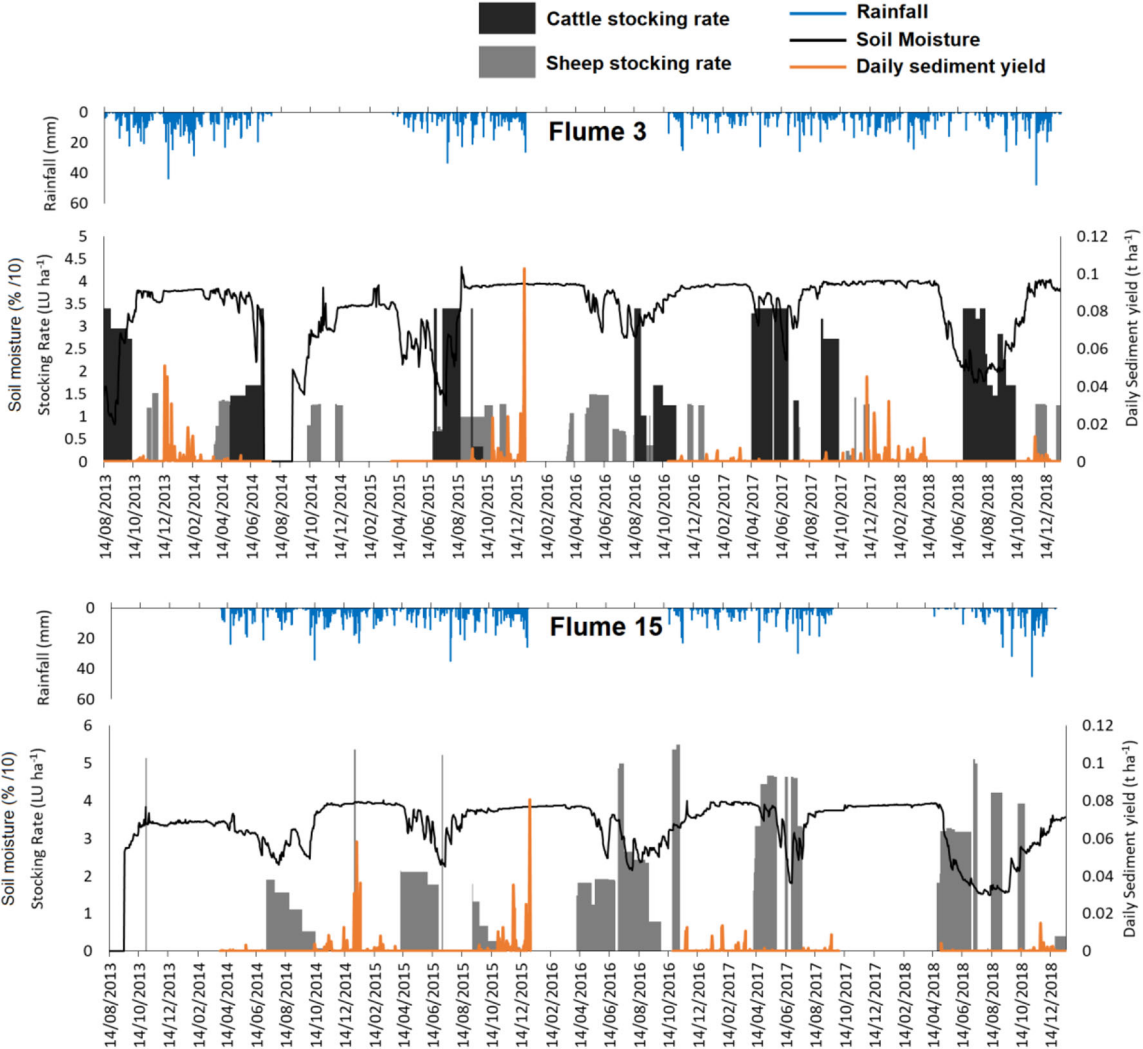
Time series of daily sediment yield, rainfall, soil moisture and livestock stocking rate for the 15 study catchments. Flow, sediment yield and rainfall data was unavailable for much of 2016

Sheep and cattle stocking rates within most NWFP field catchments are fairly consistent each year during the periods when livestock was present; however, the timing and duration of animal presence are highly variable (Fig. 2). Cattle grazing did not take place in catchments 10–15 apart from for a short (< 1 month) period in 2018 for catchments 10, 11, and 12. The timing of sheep grazing was variable; in some catchments, certain years had up to 6 months of continuous grazing such as catchment 14 in the summer of 2016. In other years, catchments were stocked on a short rotation period, with between a week and 2 months of stocking before animals were removed or stocking rates were lowered for a period of between one week and 2 months. During all winters, cattle were housed indoors as is common practice in the UK. In most of the catchments, the highest sediment fluxes occurred during December 2015. However, for catchment 14, the highest fluxes were in December 2014, and for catchment 7, they were in December 2013. All high flux periods experience heavy rainfall (Fig. 2). Importantly, no clear link between the presence of livestock and peaks in sediment yield was observed in the time series.

### Controls on long-term sediment yields

Catchment sediment yields range from 0.07 to 0.28 t ha^−1^ yr^−1^ over the ~ 6 years of monitoring (Table 1). There was a strong positive correlation (*r*^2^ = 0.90) between sediment yield and the mean suspended sediment concentration (SSC) of runoff, but no significant correlation between sediment yield and water flux. There was also no correlation between sediment yield and water yield (m^3^ ha^−1^ yr^−1^), indicating that the mean SSC of the runoff, rather than its volume, is the primary control on catchment sediment yields over a ~ 6-year timescale. The flume runoff SSC was shown by Pulley and Collins (2020) to be substantially increased through ploughing for scheduled reseeding. As ploughed periods were removed during this analysis, an alternative control such as livestock must be present.

**Table 1:** Summary data (14/08/2013–14/01/2019) for the 15 flumes

Catchment	Area (ha)	Mean slope (^o^)	Mean sheep stocking rate (LU ha^-1^)	Mean cattle stocking rate (LU ha^-1^)	Total Rainfall (mm)	Water flux (1000s m^3^)	Years of data	Mean SSC (mg l^−1^)	Sediment yield (t ha^−1^)	Sediment yield (t ha^−1^ yr^-1^)
1	4.81	5.83	0.41	0.26	3861	57.1	4.23	2.52	0.27	0.07
2	6.65	6.08	0.15	0.77	3324	31.1	3.99	6.37	0.60	0.15
3	6.62	7.29	0.29	0.64	3636	53.9	3.94	6.61	0.96	0.24
4	7.75	10.76	0.15	0.66	4081	105.3	4.62	5.77	0.80	0.17
5	6.54	12.25	0.22	0.75	3974	72.6	4.62	5.92	0.85	0.18
6	3.86	9.76	0.47	0.21	3982	34.9	4.62	4.41	0.54	0.12
7	2.60	7.54	0.55	0.17	4167	26.5	4.17	7.09	1.17	0.28
8	7.02	6.77	0.22	0.66	3493	35.1	3.99	8.36	1.03	0.26
9	7.75	8.42	0.27	0.70	3591	46.1	3.94	4.49	0.60	0.15
10	1.82	7.24	0.85	0.09	4033	14.9	4.62	3.57	0.41	0.09
11	1.76	9.71	0.75	0.06	3783	11.6	4.21	2.16	0.35	0.08
12	1.78	10.69	1.00	0.06	4491	9.6	4.62	1.83	0.39	0.08
13	1.75	7.24	0.92	0.00	4315	13.3	4.62	4.63	0.58	0.12
14	1.72	4.17	0.63	0.02	2697	7.4	3.35	4.35	0.31	0.09
15	1.54	5.32	0.80	0.00	2839	11.8	3.42	6.23	0.75	0.22

Post-plough and reseed winter periods are removed for the individual catchments subjected to such management operations

**Fig. 3:**
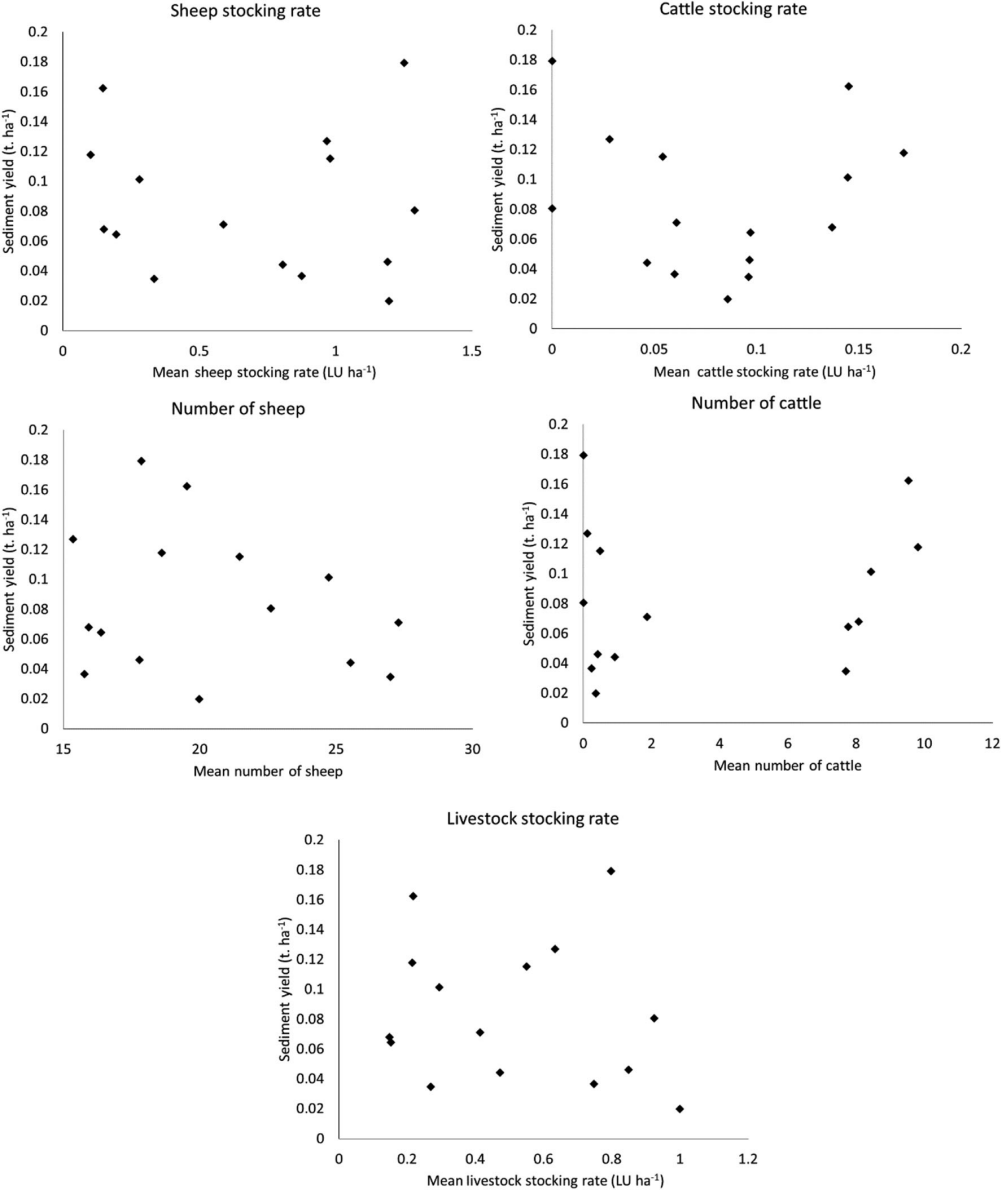
Relationships between mean annual cattle and sheep numbers, mean LU stocking rate, and sediment yields for the 2.23-year period when data was available for all catchments

To assess the long-term impact of the presence of livestock, the daily mean number of cattle and sheep and their mean stocking rate (individuals ha^−1^ day^−1^) were compared to the catchment sediment yields. The data was only compared when there was a complete day of data for every flume, with days with missing data or during post-plough and reseed winter periods removed, leaving a total of 2.23 years of data. Of the 15 catchments, six had a mean cattle stocking rate of 0.6–0.75 LU ha^−1^ (0.8–1 individuals ha^−1^) over the entire 6-year period, and the rest had a mean of 0.3 LU ha^−1^ (< 0.4 individuals ha^−1^; Fig. 3). Average beef cattle stocking rates on UK lowland forage land are 0.58 LU ha^−1^, compared with 2 LU ha^−1^ for dairy cattle (Defra 2007). There was no significant difference (*P* > 0.05, Mann-Whitney *U* test) between the sediment yields of these two groups of catchments with the 0.6–0.75 LU ha^−1^ catchments having a mean yield of 0.91 t ha^−1^ (standard deviation 0.46 t ha^−1^), and the < 0.3 LU ha^−1^ catchments having a mean yield of 0.80 t ha^−1^ (standard deviation 0.52 t ha^−1^). It therefore is apparent that there is little observable long-term impact of cattle grazing on long-term sediment yields (Fig. 3). Similarly, mean sheep stocking rates range from 0.14 to 0.99 LU ha^−1^ (2.1–12.9 individuals ha^−1^), and there is no significant correlation between the number of sheep, sheep stocking rate, and the catchment sediment yields (Fig. 3). The mean stocking rate on UK lowland sheep farms is 5.9 ewes ha^−1^ (0.65 LU ha^−1^; 2016–2017) (Fogerty et al. 2018). This analysis was repeated using the stocking rates for the entire monitoring period rather than just the rates for the 2.23 years of complete sediment yield data, and again, no significant correlations with sediment yield were found for either sheep or cattle.

### Controls on monthly and winter sediment yields

**Fig. 4:**
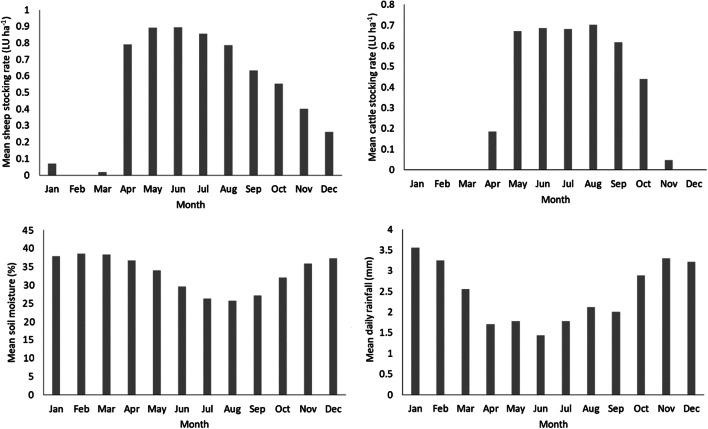
Monthly mean sheep and cattle stocking density, soil moisture, and rainfall

There are strong correlations between monthly water flux and sediment yield as well as mean SSC and sediment yield when the data for all flumes was combined (Table 2). Rainfall and mean soil moisture were also significantly positively correlated with sediment yield, which reflects the fact that wetter months have greater flows and sediment yields than drier months. Water flux and mean SSC were correlated, with an *r*^2^ of 0.72, indicating that there is a moderate amount of variance in SSC, which is not accounted for by increased flow and is likely related to the erodibility of the grazed fields and the availability of sediment for mobilisation. Mean monthly cattle and sheep stocking rates were, however, not correlated with sediment yield. It should be considered though that whilst some sheep remained in fields for the entire year, there was a significant decrease in animal numbers over autumn months with very few animals left present during winter and early spring (Fig. 4). Similarly, cattle were not present in fields from mid-autumn to winter months in conjunction with best practice management. As a result, during wetter months when sediment yields are highest, livestock will be largely absent, and a significant correlation would not be expected.

**Table 2:** Spearman rank correlations (*r*^2^)

	Sheep stocking rate	Number of sheep	Cow stocking rate	Number of Cattle	Soil Moisture	Water Yield	Rainfall	Mean SSC	Sediment Yield
Sheep stocking rate	-	0.90	*0.02*	*0.02*	*0.07*	*0.07*	*0.01*	*0.06*	*0.05*
Number of sheep	-	-	*0.00*	*0.00*	*0.07*	*0.05*	*0.00*	*0.06*	*0.05*
Cow stocking rate	-	-	-	0.97	*0.08*	*0.03*	*0.01*	*0.02*	*0.03*
Number of cattle	-	-	-	-	*0.07*	*0.02*	*0.01*	*0.01*	*0.03*
Soil moisture	-	-	-	-	-	0.49	0.09	0.41	0.45
Water yield	-	-	-	-	-	-	0.38	0.72	0.77
Rainfall	-	-	-	-	-	-	-	0.36	0.45
Mean SSC	-	-	-	-	-	-	-	-	0.90

Values in italics are negative before being squared, bold text indicates a significant relationship (*P* < 0.05)

**Fig. 5:**
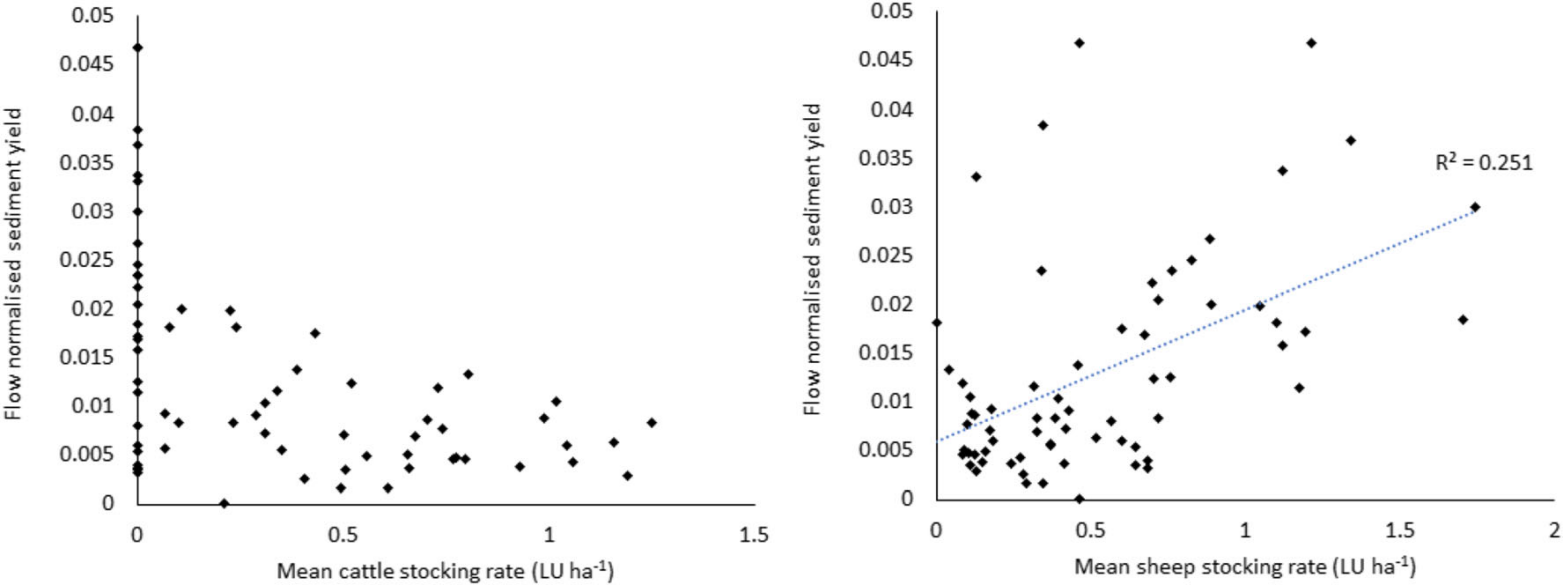
Flow normalised sediment yield plotted against livestock stocking rates for individual catchments

It was determined if soil disturbance caused by grazing in summer and autumn months resulted in an increased sediment yield in winter months. For this analysis, the winter of 2018–2019 was removed as data was not available past the 14 January 2019. The only significant correlation (*P* < 0.05) found between livestock numbers and stocking rates throughout the preceding 1 April to the 31 of March, and winter sediment yield normalised to water flux (1 November–31 of March) was a low *r*^2^ of 0.25 with average sheep stocking rate (Fig. 5).

**Fig. 6:**
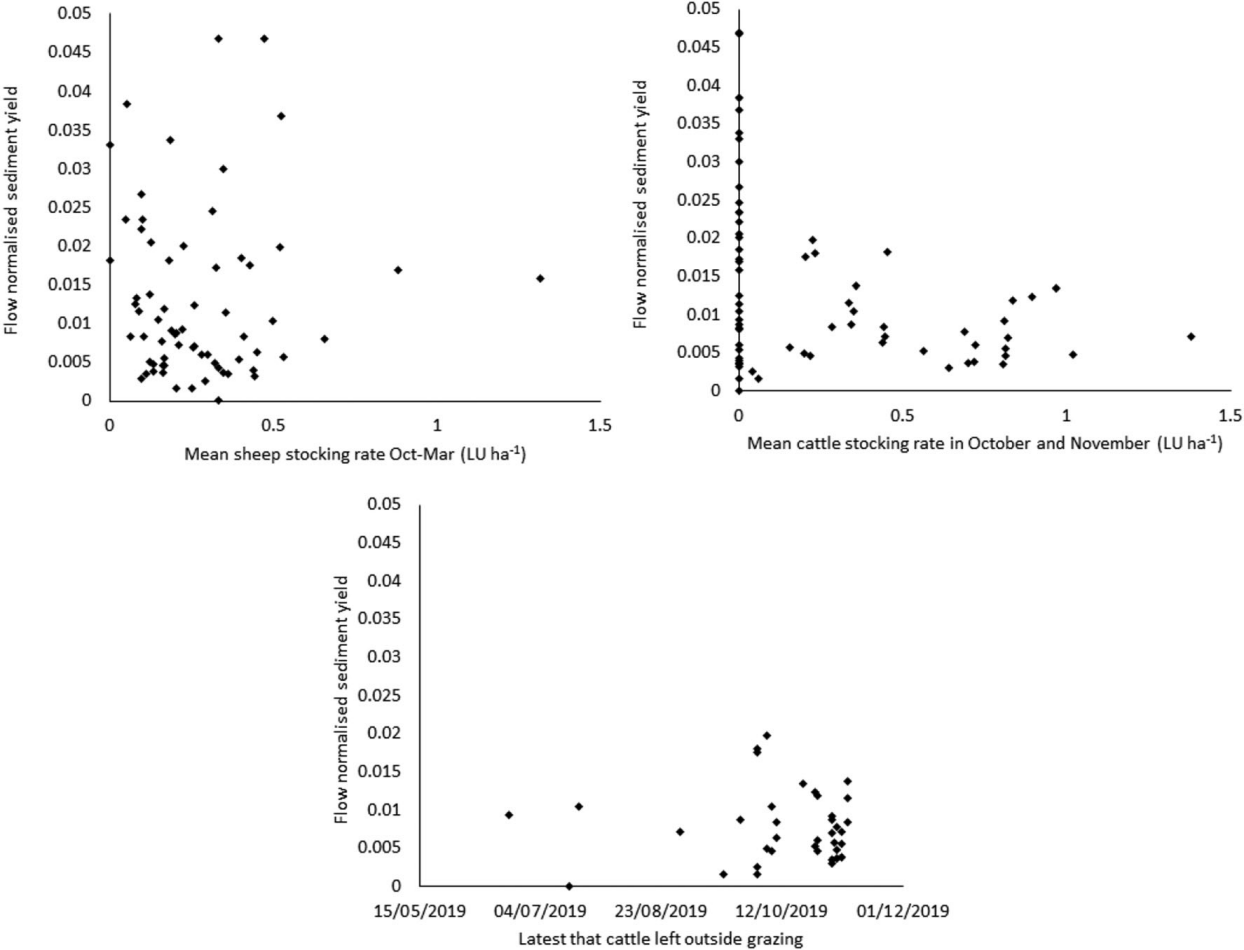
Flow normalised sediment yield plotted against the mean sheep stocking rate from October–March, the mean cattle stocking rate in October–November, and the latest date cattle were left outside grazing

Of particular interest are the months of October and November where rainfall and soil moisture increase but cattle often remain present in the fields (Fig. 4). There are, however, also no significant correlations between the timing of cattle and sheep grazing into autumn and winter months and winter sediment yields normalised to water flux (Fig. 6). When examined on an individual catchment basis, none shows a clear indication of an increase in winter water flux-normalised sediment yield when stocking rates were higher (Supplementary Fig. 1).

### Field use by cattle and their impacts on soil cover and bulk density

**Fig. 7:**
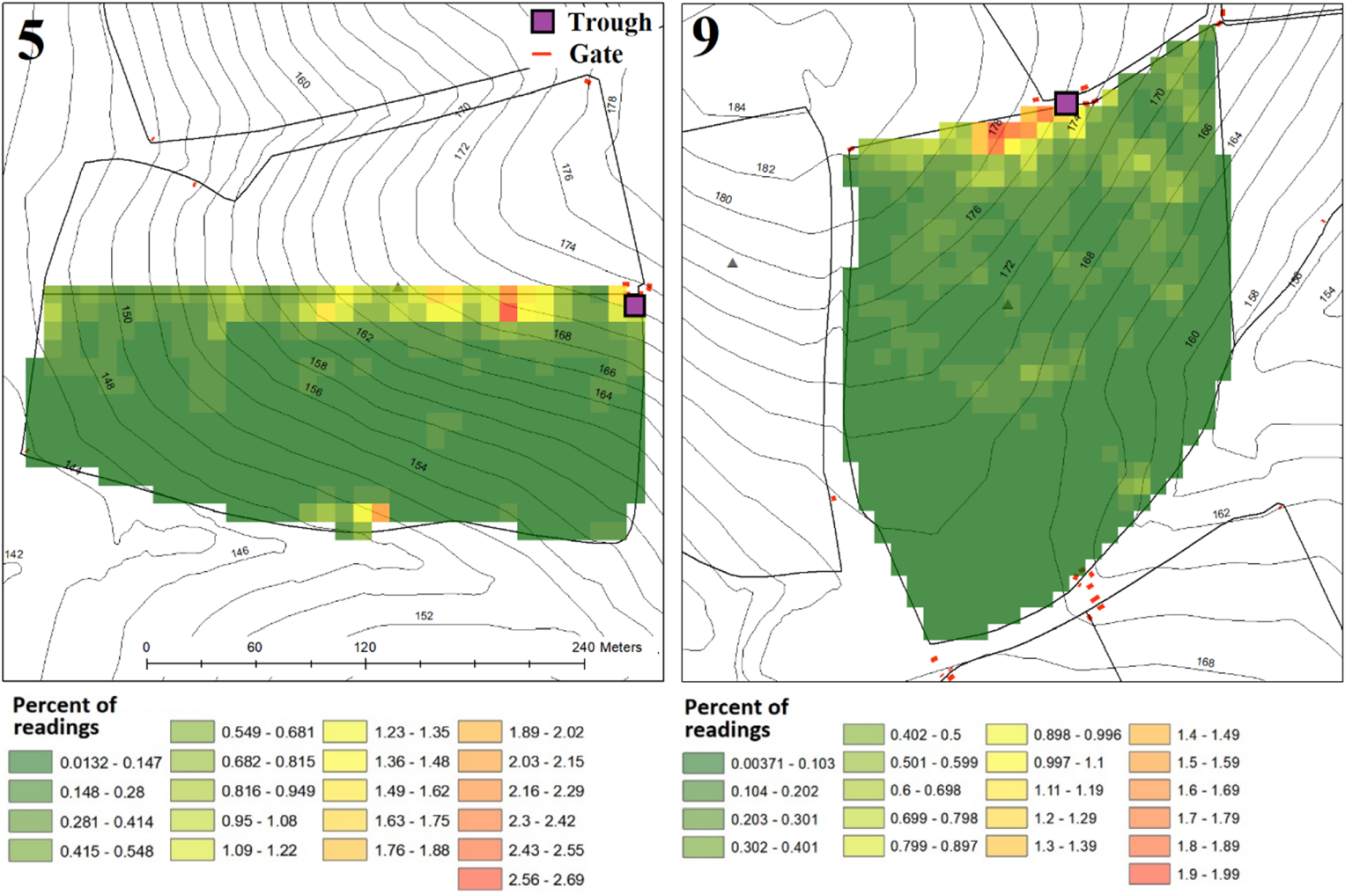
The percentage of cattle GPS readings recorded in each 10 m × 10 m cell of catchments 5 and 9

The GPS tracking of cattle movement in the bottom half of catchment 5 and the whole of catchment 9 during a week in the summer of 2018 show uneven use of the respective field areas (Fig. 7). For catchment 5, 50% of cattle time was spent in just 11% of the field area, whereas in catchment 9, 50% of time was spent in 14% of the field area. The tracking data confirmed a clear tendency for the cattle to congregate preferentially along one fence in each field. In both fields, this was at the highest elevation and in proximity to water troughs. Daytime temperatures (6 am–6 pm) during the monitoring period were mild at a mean of 20.7 °C for catchment 5 and 15.2 °C for catchment 9 and little rainfall occurred (0 mm in catchment 5 and 1.6 mm in catchment 9).

**Fig. 8:**
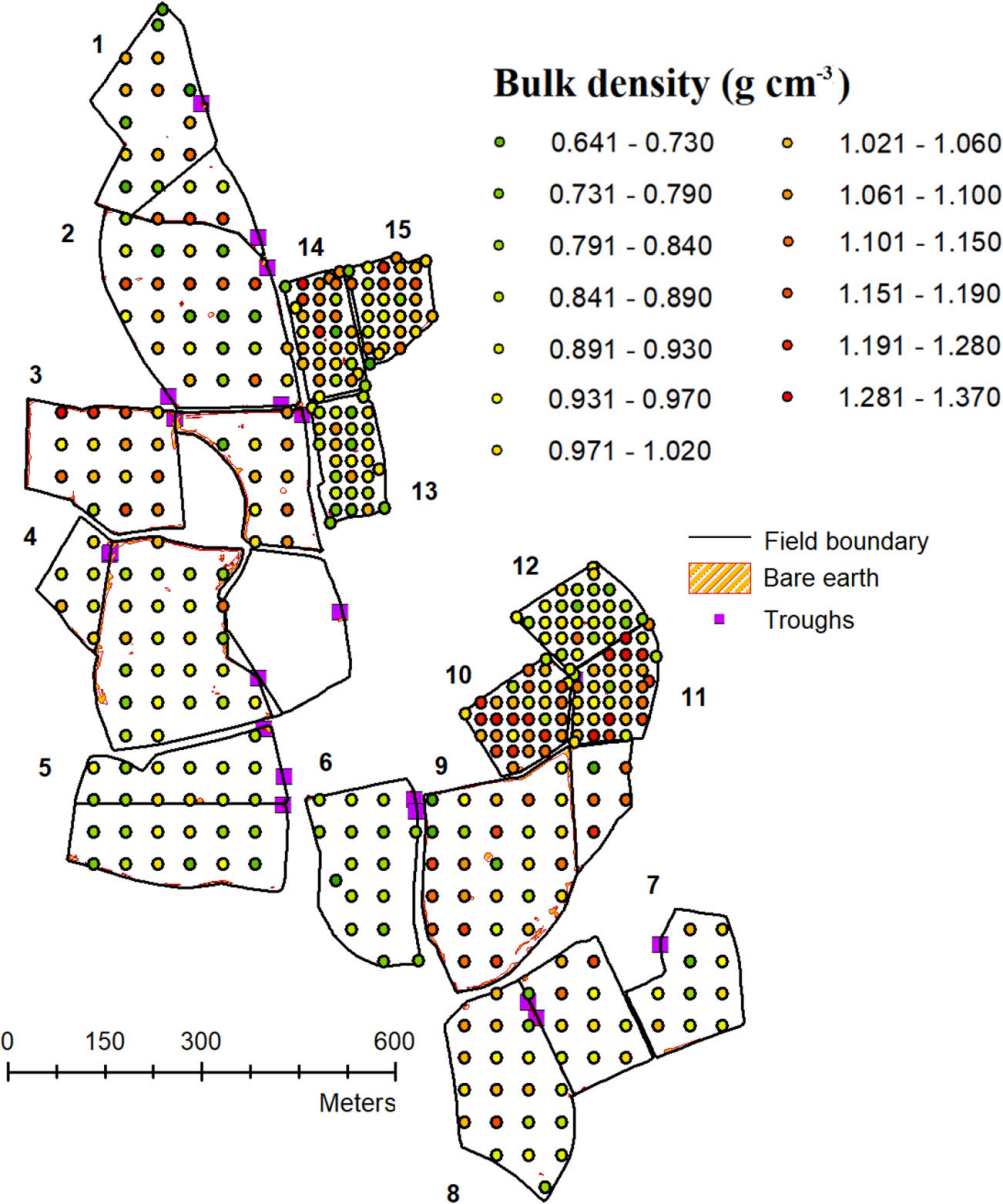
Areas of visually damaged soil identified using an aerial photograph taken in mid 2016 and soil bulk density (g cm^−3^) survey (2016) data

The mapping of poached areas of soil (Fig. 8) lacking vegetation cover in the summer of 2016 also showed that soil damage was primarily located in narrow strips along fences and by gates and troughs ,and this was confirmed by visual observations in subsequent years. Of the catchments with the largest areas of visually damaged soils, catchments 9 and 4 had the most cattle present when the aerial photograph was taken; however, catchment 2 also had cattle present but did not show the same extent of soil damage by surface poaching. Of note here is catchment 3 which had significant areas of bare soil but did not contain cattle during 2016. It did, however, have a high sheep stocking rate, which given the large size of the field, equated to many individual sheep present (*n* = ~ 150). It is therefore likely that a preference of cattle to congregate along or near fences, troughs, and gates is causing sward loss which is highly localised to a narrow strip along field margins. It is also notable that larger fields generally had a greater area of damaged soil than smaller ones which is likely due to these fields being preferentially used for cattle grazing as well a larger total number of animals being present which are all preferentially congregating within a small area of the field replicating very high stocking density within that area.

When surveyed in July 2016, there was considerable variability in soil bulk density within the 15 catchments. Bulk density was not observed to increase close to fences where most of the visually damaged soil was located. However, sampling is not specifically targeted to assess edge-of-field compaction, so no samples are available in the narrow most trampled areas, which were typically less than 1-m width (Fig. 8). As part of a study conducted in October 2020, nine bulk density samples were retrieved from heavily poached areas around gates, fences, and troughs in a field 500 m to the northwest of those examined in this study. The mean bulk density of these was 1.42 g cm^−2^ (standard deviation 0.14 g cm^−2^) which was significantly higher than any sample measured in the NWFP 2016 spatial survey which did not target these areas (Morten et al. 2020, unpublished data). Within the study site, there was no significant difference between mean soil bulk density in the catchments where cattle were normally present (catchments 2, 3, 4, 5, 8, and 9) (mean 0.97 g cm^−3^; standard deviation 0.14 g cm^−3^) and those catchments where cattle were rarely present (mean 0.99 g cm^−3^; standard deviation 0.15 g cm^−3^). There was also no significant relationship between sediment yield and mean soil bulk density in the 15 catchments.

## Discussion

No clear impact of livestock numbers, stocking rate, or grazing season length on sediment yield was identified in the field scale catchments studied. Whilst the preference of cattle to congregate around particular fences and troughs is causing vegetation loss, compaction, and shearing of the soil, this effect was limited to a very small proportion (< 5%) of the total field areas. Observations reported by Pulley and Collins (2019) noted that on the NWFP, concentrated saturation-excess overland flows over heavily poached soils along field margins were not entraining high concentrations of sediment, leading to the conclusion that sediment mobilisation is field-wide in conjunction with raindrop-impacted saturation-excess overland flow. Subsequent observations have noted two instances where trampled field margins were experiencing disproportionate sediment loss. However, this is uncommon and limited to small (< 5 m^2^) areas. Because of the clayey soils present and their resistance to erosion from overland flows alone, and indeed the erosion buffering effect of runoff depths exceeding raindroplet diameters, any decrease in water infiltration caused by soil compaction during livestock grazing has likely not resulted in a substantial increase in sediment yield. Soil surface poaching and removal of the grass sward through grazing present one mechanism by which livestock could increase erosion rates since more raindrops would impact the soil surface directly. However, such an effect was not observed on the sediment yields discussed herein, possibly due to a combination of pre-existing best management stocking rates and appropriate grazing season duration, and a tendency of the livestock to preferentially overuse only a small area (~ 10%) of the fields in question.

The lack of a detectable impact of livestock grazing under best management on sediment yields presents a significant contrast to when some of the same fields were ploughed and reseeded as part of routine sward management and large increases in sediment loss were observed (Pulley and Collins 2020). In the case of the latter, it was found that a mean of 28.8% of ~ 6-year total sediment flux took place during the immediate post-plough winters despite them only covering a mean of 10.9% of the monitoring period. When two fields were ploughed in wet autumn months, the increase in sediment yield was far higher at up to 56% of the total ~ 6-year sediment yield occurring during two winter periods. Whilst the new study reported herein cannot conclude that livestock are having absolutely no impact on sediment losses, our analysis suggests that other factors such as variability in rainfall or field morphology must have much more of an impact than that of continuously stocked livestock grazing under best management. Previously published research elsewhere has shown an increase in erosion rate associated with intensive livestock grazing (Branson and Owen 1970; Gifford 1975; Lusby 1970; Bilotta et al. 2010), indicating that the observed lack of impact is likely to be dependent on local factors and especially the erosion-resistant clayey soil texture (Dunne and Black 1970; Anderson and Burt 1978; Horn et al. 1995).

There is currently a lack of evidence regarding the efficacy of many on-farm management interventions at landscape scale (Kay et al. 2009; McGonigle et al. 2014; Randall et al. 2015). This presents a challenge when trying to quantify the impacts of improved management from a cost-benefit perspective, and as such, there are increasing attempts to optimise the uptake of mitigation measures accordingly (Haygarth et al. 2007; Gooday et al. 2014). At present, most catchment/agricultural advisors will assess potential pollutant sources through a rapid walkover visual assessment of soil damage and perceived risk to water quality. Whilst in some cases, this will be effective, in many situations, actual sediment sources may not correspond well to visually perceived sources of the problem. For example, Buddulph et al. (2017) showed that remediating a heavily degraded farm track failed to deliver a significant change in sediment provenance even at a farm scale due to it only covering a small proportion of the total catchment area and other sediment sources being dominant. The results presented herein suggest that mitigation options applied based upon a visual assessment of damage to soils by livestock on the NWFP are unlikely to result in a substantial further reduction in sediment loss over and above the benefit associated with best practice grazing management comprising appropriate stocking rates, grazing season length/overwinter housing, and removal during wet weather.

In association with the European legislation for water quality and the ambition to reduce the detrimental impacts of modern intensive farming, current agricultural policy in the UK combines regulation, advice, and incentivisation to drive the uptake of best practice. In England, the Catchment Sensitive Farming (CSF) initiative, which is run in partnership by the Environment Agency and Natural England, has engaged with 34% of the national farmed area. Through this initiative, officers deliver free advice to farmers aimed at reducing pollutant losses to water and air and matched grants through the Countryside Stewardship scheme are also available in priority areas (Natural England 2019). Through CSF, there is a high uptake of advice specifically related to livestock management. For example, there is a ~ 80% uptake rate when ‘reducing livestock stocking densities when soils are wet’ is recommended as a best management intervention, and an uptake rate of ~ 70% when ‘reducing the length of the grazing day or season when weather conditions and soils are unfavourable for avoiding poaching’ are recommended (Natural England 2019). There is also close to a 60% uptake rate when ‘moving feeder and water troughs regularly or onto a hard standings’ is advised. When combined, these options excluding avoiding advice to reduce poaching represent 8.8% of measures implemented by farmers engaged by the CSF initiative. Therefore, best practice interventions aimed at reducing pollutant losses associated with livestock grazing are being widely applied across England. The annual costs associated with these specific interventions have been estimated to be: reducing livestock stocking rates when soils are wet = £2.43 ha^−1^ (operational cost only); reducing the length of the grazing day or season when weather conditions and soils are unfavourable = £1.60 ha^−1^ (dairy) £1.43 ha^−1^ (beef) (both operational costs only); and moving feeder ring and water troughs regularly or onto a hard standing = £12.53 ha^−1^ (operational cost only) (Gooday et al. 2014). The results of our study here, however, suggest that only the former two options are likely to be cost-effective as part of best practice in environmental settings similar to the NWFP. This is because our analysis shows that the implementation of these two interventions means that there is no detectable impact of livestock presence on sediment loss.

## Conclusions

The results of this study suggest that in temperate lowland grazing landscapes with erosion-resistant clayey soils and best practice grazing management focussed on appropriate stocking rates and duration of grazing season linked to the onset of increased rainfall and soil moisture content, the presence of livestock does not substantially elevate sediment loss from grazed grassland. The common practice of housing cattle indoors during wet winter months in the UK is likely to be a major contributing factor to this lack of observable impact. As such, further mitigation measures such as periodically moving feeder rings or installing concrete bases for water troughs are unlikely to deliver further benefits in reducing sediment losses. Clearly, this lack of an impact from livestock grazing would not be the case if best practice grazing management was not implemented and stocking densities were higher and outdoor wintering used regardless of elevated soil moisture contents. In terms of sediment losses on the NWFP, previous research has shown that scheduled ploughing and reseeding represent the dominant risk factor and as such should be managed carefully as part of the routine operations on lowland grazing farms. Whilst the implementation of best practice grazing management means there is no discernible impact of livestock presence on sediment loss, it is important to acknowledge that livestock grazing will inevitably be associated with some alternative unintended consequences including gaseous emissions which need to be managed as part of mitigation strategies carefully designed to take explicit account of multiple environmental risks arising from modern farming.

## Supplementary Information

ESM 1 (DOCX 671 kb)

## Data Availability

Data is available from the North Wyke Farm Platform Data Portal at https://nwfp.rothamsted.ac.uk/ Not applicable
