# Supplementary material for: Does cattle and sheep grazing under best management significantly elevate sediment losses? Evidence from the North Wyke Farm Platform, UK
Source: J Soils Sediments. 2021 Mar 13;21(4):1875–89. doi: 10.1007/s11368-021-02909-y (PMC8550719; doi:10.1007/s11368-021-02909-y)
Supplement: Supplementary file 1 — (DOCX 671 kb) [file 11368_2021_2909_MOESM1_ESM.docx]

# Supplementary information


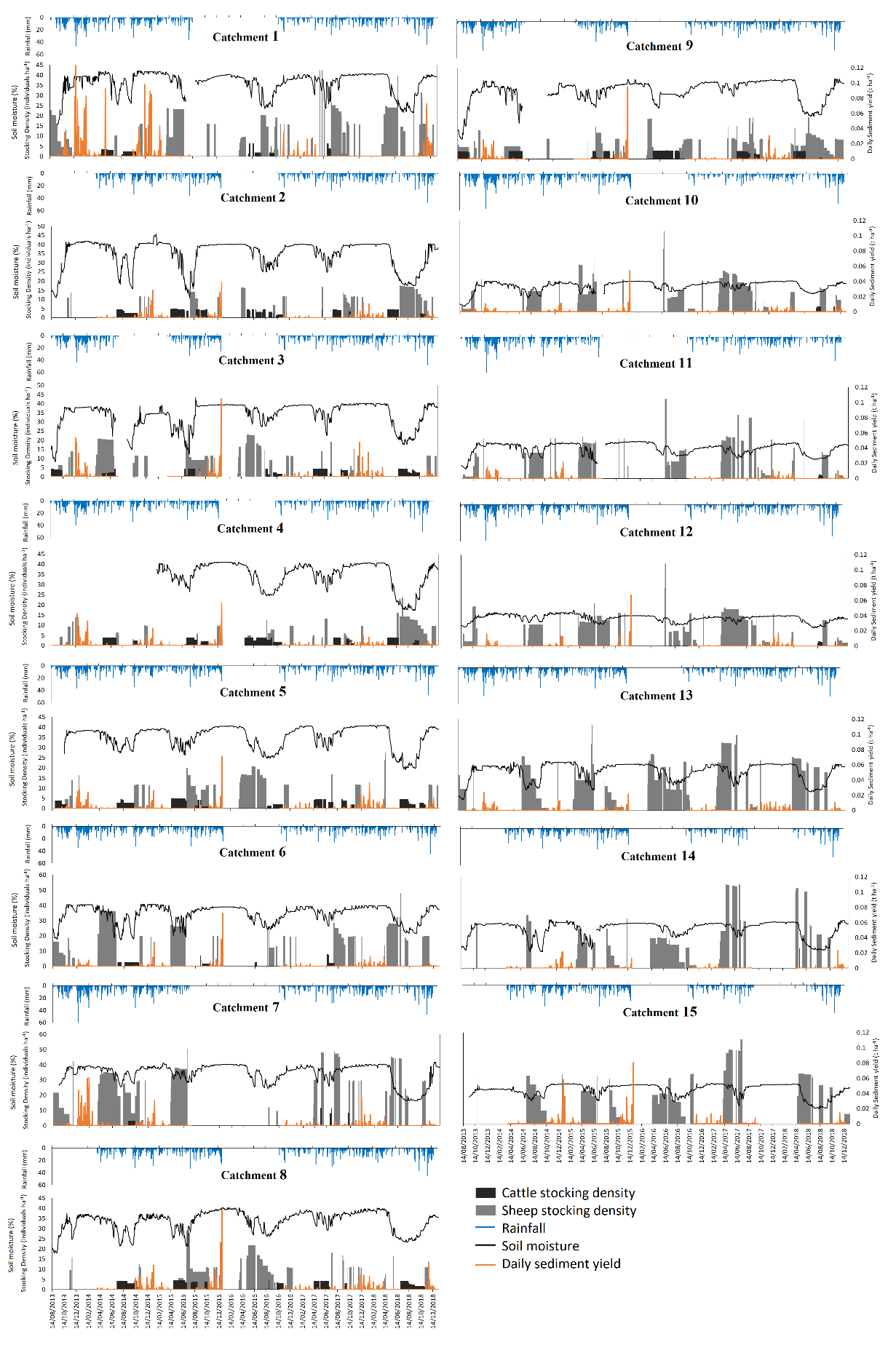


Fig.S1: Time series of daily sediment yield, rainfall, soil moisture and livestock stocking rate for the 15 study catchments. Flow, sediment yield and rainfall data was unavailable for much of 2016


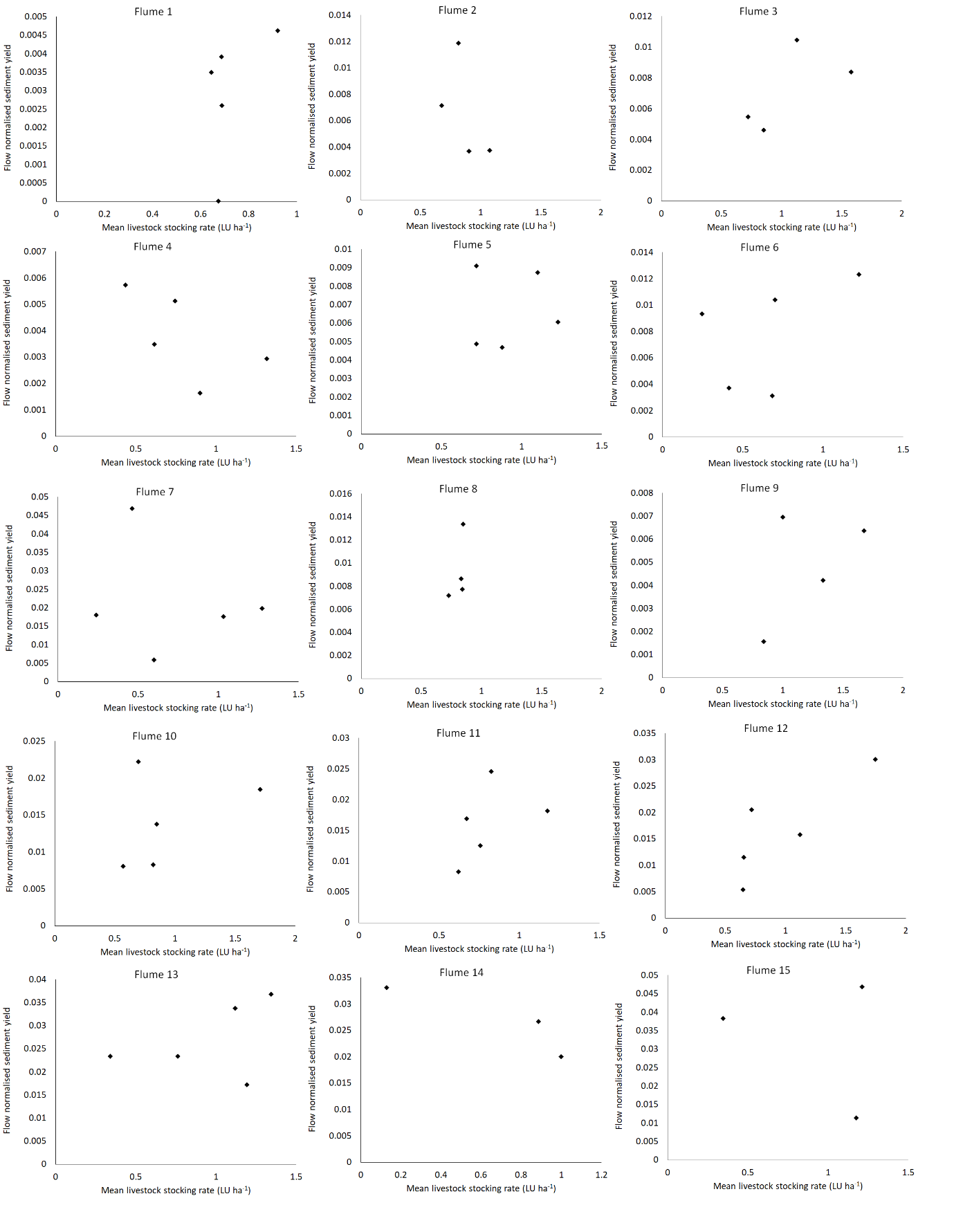


Fig.S2: Water flux-normalised winter sediment yield plotted against mean livestock (cattle + sheep) stocking rate (1^st^ April – 31^st^ March).
